# Guidelines versus evidence: what we can learn from the Australian guideline for low-level laser therapy in knee osteoarthritis? A narrative review

**DOI:** 10.1007/s10103-020-03112-0

**Published:** 2020-08-08

**Authors:** Roberta Chow, Ann Liebert, Sharon Tilley, Gregory Bennett, C. Philip Gabel, Liisa Laakso

**Affiliations:** 1 Faculty of Medicine and Health, Brain and Mind Centre, University of Sydney, 94 - 98 Mallett Street, Camperdown, New South Wales 2050 Australia; 2 Photomolecular Research, Sydney Adventist Hospital, Wahroonga, New South Wales Australia; 3 Faculty of Medicine and Health Sciences, University of Sydney, Camperdown, NSW Australia; 4 Private Practice, Lymphoedema and Laser Therapy, 140 Payneham Rd, Stepney, South Australia 5069 Australia; 5 Sydney Adventist Hospital, Wahroonga, New South Wales 2076 Australia; 6 Private Research, Coolum Beach, Sunshine Coast, 4573 Australia; 7 Menzies Health Institute Queensland Griffith University, Gold Coast, Australia; 8 Mater Research, South Brisbane, Australia

**Keywords:** Photobiomodulation, Knee osteoarthritis, Guidelines, Low-level laser therapy

## Abstract

This narrative review analyses the Australian Guideline (2018) for the treatment of knee osteoarthritis (KOA) developed using Grading of Recommendations Assessment, Development, and Evaluation (GRADE) methodology. The Guideline recommended against the use low-level laser therapy (LLLT). Why this conclusion was reached is discussed in this review in the context of evidence provided in other systematic reviews, the latest of which was published in 2019 and which provided strong support for LLLT for knee OA. We evaluated the reference list cited for the recommendation “against” LLLT and compared this with reference lists of systematic reviews and studies published before and after the publication date of the Guideline. Eight randomised controlled trials (RCTs) of LLLT were cited in the Guideline the latest of which was published in 2012. There were seventeen additional RCTs, five of which together with one systematic review were located in the year of publication, 2018. The most recent systematic review in 2019 included 22 RCTs in its analysis. Discordance with the levels of evidence and recommendations was identified. Although GRADE methodology is said to be robust for systematically evaluating evidence and developing recommendations, many studies were not identified in the Guideline. In contrast, the latest systematic review and meta-analysis provides robust evidence for supporting the use of LLLT in knee OA. The conflict between guidelines based on opinion and evidence based on meta-analysis is highlighted. Given the totality of the evidence, we recommend that the Australian Guideline should be updated immediately to reflect a “for” recommendation.

## Introduction

Clinical practice guidelines are used by health professionals to promote evidence-based practice, but controversies exist regarding transparency of recommendations, possible bias and how to keep guidelines current when new information becomes available. GRADE (Grading of Recommendations Assessment, Development, and Evaluation) methodology which is described as a “a transparent framework for developing and presenting summaries of evidence and provides a systematic approach for making clinical practice recommendations” has been developed to address such areas of concern [1]. The GRADE process begins with an explicit question and all the available evidence is collected and summarised. GRADE methodology then details the criteria for rating the quality of the evidence and developing graded recommendations [2]. Subjective consideration of the values and preferences, resource implications and desirable versus undesirable consequences of options are part of the GRADE-based assessment setting it apart from other evidence reviews.

In 2018, the Royal Australian College of General Practitioners (RACGP) published a Guideline for the management of knee and hip osteoarthritis (OA) (referred to as “the Guideline” herein) [3]. For knee OA, the RACGP Guideline reviewed a large range of treatments including low-level laser therapy (LLLT). This is now an outdated term for “photobiomodulation” (PBM) which now replaces LLLT as the MESH term. For simplicity and consistency with the documents cited, this will be referred to as LLLT within the Guideline. The recommendation for LLLT was “conditional against”. In this narrative review, we examine how GRADE methodology was applied to the literature search on which recommendations were based; the currency of the evidence; the rationale; and validity of the recommendations and how these conflict with more recent evidence.

## Methods

Firstly, we outline the GRADE procedures used by the Working Group to arrive at the “conditional against” recommendation for the use of LLLT in knee OA [4].

### Identification of studies

The first step in the GRADE process is to identify studies on which GRADE quality of evidence assessment is based. The methods used by the Working Group followed accepted principles. The PICO (Patient/Problem/Population, Intervention, Comparison/Control/Comparator, Outcome) questions developed by the Working Group were similar for all of the interventions investigated. For LLLT, the PICO question sought to identify “benefits and harms of laser in the management of patients with knee OA” (p. 67). Importantly, search terms such as “laser therapy”, “photobiomodulation” and “LLLT” did not appear in the search strategy [4].

To confirm that all available RCTs were identified within the time frame prior to the Guideline publication, we conducted a search of all published systematic reviews of LLLT in knee osteoarthritis (KOA) to 2019, hand-searched the reference list in each review and conducted an additional search of LLLT literature to identify any additional RCTs.

### Analysis of the quality of the evidence profile

The next step undertaken by the Working Group was a quality assessment of the eight identified studies and formulation of the evidence report incorporating the GRADE evidence profiles categorised as high, moderate, low and very low. The Working Group did not perform any meta-analyses and no list of excluded studies was supplied. For LLLT, the evidence was rated by the Working Group as “low” though the Guideline stated that there are “clinically meaningful benefits in short-term pain and function (up to three weeks)” (p. 38). Considering this positive statement, we proceeded to a more detailed analysis of the recommendations.

### Analysis of the recommendations

A final step in the GRADE process is the formulation of recommendations designated as (i) “strong recommendation ‘for’”, (ii) “conditional recommendation ‘for’”, (iii) “neutral”, (iv) “conditional recommendation ‘against’” and (v) “strong recommendation ‘against’”. The recommendations are based not only on the quality of the evidence but also on subjective assessments of the balance between benefits and harms, values and preferences as perceived by the Working Group, and resources for the options under consideration. We compared the evidence and rationale for the GRADE recommendations for a random sample of other non-pharmacological and pharmacological treatment options in the Guideline.

## Results

### Literature review of the guideline

Eight RCTs for LLLT were cited in the Guideline [5–12], the last of which was published in 2012 though the Guideline was published in 2018. We identified six systematic reviews and meta-analyses [13–18], the latest of which was published in 2019. Many of the references we located were available prior to publication of the 2018 RACGP Guideline but were not included in the Guideline analysis. No criteria for excluding studies were identified and therefore, the rationale for selection of the included studies is unclear.

To date, we have located a total of twenty-five studies of LLLT for KOA or knee pain, seventeen of which were not cited in the Guideline though were available up to 2018 [19–35]. One study has not been considered as it was a study using laser acupuncture, though had been included in several of the systematic reviews [36].

Of these, there were seven double-blind randomised studies comparing LLLT with placebo (sham laser) [19–24, 35]. Of these seven studies, five demonstrated both clinically and statistically significant results favouring LLLT. One double-blind RCT treated “knee pain” rather than KOA though logically, this study should be included as pain is a primary symptom of KOA and the ages of the patients included in the study were older than 50 years [28]. One of the additional studies compared LLLT with transcutaneous electrical nerve stimulation (TENS), with LLLT having a statistically significant benefit over TENS [25]. Several randomised placebo-controlled single-blind trials used various experimental designs: for example, sham laser versus active laser [33]; high-dose laser + exercise versus low-dose laser + exercise versus sham laser + exercise [27]; high-dose laser versus low-dose laser versus exercise alone [34]; LED + exercise versus LLLT + exercise [26]; laser versus sham laser [30]; laser versus physiotherapy [31]; laser + exercise versus sham laser + exercise [32].

Since the Guideline was published, several additional studies which further extend the evidence base regarding LLLT and KOA were identified: a double-blind RCT of LLLT + exercise compared with sham LLLT versus exercise at 3 and 6 months after a course of 10 treatments and showed a significant decrease in analgesic rescue medication in the laser group at 6 months after treatment (*p* < 0.001) [37]; a double-blind RCT showed LLLT + exercise significantly reduced pain compared with exercise + sham LLLT and exercise alone (*p* < 0.05) [38]; a single-blind randomised study showing maximal improvement in gait parameters with a laser (808 nm) + exercise group (*p* = 0.003) [39]; a single-blind study using ultrasound + laser + exercise showed a significant improvement in pain and function in the combined ultrasound + laser group with or without exercise (*p* < 0.05) [40]; a double-blind RCT comparing laser versus physiotherapy versus exercise alone showed significant improvement in pain and function in the laser group compared with other groups (*p* < 0.001) [41]; and a systematic review of studies using high intensities of LLLT showing a consistent benefit over placebo for knee OA [42].

### Systematic review and meta-analysis 2019

Since the publication of the Guideline, a systematic review and meta-analysis included 22 RCTs (*n* = 1063) and found strong evidence for the reduction in pain and disability with LLLT for KOA with no adverse effects [18]. Several studies not previously identified were included in the review [43–49]. Of these studies, four used a laser to acupuncture points. Pain was significantly reduced compared with placebo at the end of therapy: 14.23 mm VAS improvement compared with placebo at the end of therapy and 23.23 mm VAS improvement during follow-ups 2–12 weeks after the end of therapy. Improvement peaked at 2–4 weeks after the end of therapy. Disability was also significantly reduced. When recommended doses of laser were used, pain was significantly reduced at the end of treatment (18.271 mm VAS) and during follow-up 2–12 weeks later (23.23 mm VAS). Pain reduction peaked during 2–4 weeks at follow-up (31.87 mm VAS). LLLT using 4–8 J per point with 785–860-nm laser and 1–3 J with 904-nm laser was found to be effective.

### Comparison of Guideline evidence profile between modalities and recommendations

When comparing the examples of listed interventions in the Guideline with the same level of “low” evidence as LLLT, it is apparent that while evaluation of harm was one criterion for developing and differentiating recommendations, there was inconsistency in why one “harmless” modality such as massage was recommended and LLLT which the Guideline described as having no adverse effects was recommended “against”.

## Discussion

Knee OA is common and costly, both to the individual due to multiple associated comorbidities, including stroke and cardiovascular disease [50], and to health systems due to the economic burden it generates. Many of the therapies outlined in the Guideline have low or very low levels of evidence, are compromised by short-term benefit only, serious side effects when used long term, and are invasive or addictive. The recommended treatments require a long-term commitment to weight loss, psychological support and regular exercise, all desirable goals but often unachievable in the “real world” most likely due to matters associated with adherence [51]. There is clearly a need for other treatment options in KOA as a “cure” for the disease currently appears elusive.

Photobiomodulation is a therapy used pragmatically for OA for 40 years with the first RCT of LLLT in KOA performed in 1987 [19]. Mechanisms for statistically and clinically significant benefits of LLLT include anti-inflammatory effects, in some studies equivalent to that of drugs, optimising tissue repair, enhancing muscle function and modulating nociceptor activity to relieve pain via neural blockade. LLLT offers patients a non-drug, non-invasive treatment with minimal side effects which can be delivered in different clinical settings including with home-use devices [52].

Of interest is the grouping of LLLT with “other electrotherapy” modalities including shockwave, ultrasound and interferential therapy. This is a meaningless legacy grouping that simply classes the machine as having a switch that can be turned on and off. *What* comes out of the “switched device” is critical and differs vastly across the grouping listed in the Guideline. The fundamental physics of LLLT as electromagnetic energy bears nothing in common with shockwave therapy or ultrasound with the latter therapies generating pressure waves in tissue with no electromagnetic energy characteristics. Similarly, interferential is electrical energy with completely different characteristics from electromagnetic energy.

Several other factors have also contributed to the Guideline inadequately evaluating LLLT.

Firstly, the limited number of RCTs cited in the Guideline, eight, when there were seventeen other published RCTs within the Guideline timeframe means that the literature search method was not comprehensive. We acknowledge that the quantity of RCTs does not override their quality; however, as there was nothing to indicate whether other RCTs were found and excluded, we have to assume that the other RCTs were simply not identified. This may in part be due to the failure of the search strategy to include any of the usual synonyms for “laser” such as “PBM”, “photobiomodulation” or “laser therapy”. A list of exclusion criteria and excluded studies would have created greater transparency and should have been included if available. It is not clear from the technical document if studies were systematically as undertaken thus failing to adhere to the requirements of the GRADE process that all appropriate RCTs should be identified. Also, of note is that the latest of the cited papers was published in 2012 though the Guideline was published in 2018. The difference is stark between the latest systematic review [18] with 22 studies which show both clinically and statistically significant pain relief as well as improvement in disability scores. This factor demonstrates the lengthy time frames associated with Guideline development by committee and the folly of publishing Guidelines that are not reviewed and updated regularly.

A second aspect to be reconciled was why the quality of evidence did not appear to be used in a consistent manner when making recommendations. Some of the recommended treatments had equally “low” levels of evidence as LLLT and more cited evidence, e.g. compared with massage, but LLLT was not recommended. To understand this decision, we were led to consider a third issue of “values and judgements” which are features of GRADE methodology differentiating it from other systematic assessments of evidence. GRADE recommendations are based not only on the quality of the evidence but also on subjective assessments of the balance between benefits and harms, values and preferences as perceived by the Working Group. Such subjective assessments reverse the “pyramid” of evidence in which level 1 evidence from meta-analysis, which was formerly considered the strongest level of evidence, is now less important [53]. Values and judgements have become subject to “consensus” which was at the base of the evidence pyramid as the lowest level of evidence. In practical terms, this means that treatments with “very low” or “low” levels of evidence can be recommended if harms are judged as low and there is a clinical benefit when a group agrees. In the 2018 Guideline, the subjective nature of this recommendation is illustrated by the opening of the sentence “We believe……” when describing the reasons for an “against” recommendation. While a subjective assessment using “values and judgements” may be necessary for guidelines, it must be based on all the available evidence. While meta-analyses can also give false measures of benefit, consensus statements based on limited evidence may be equally flawed.

The subjectivity of the process raises the question as to what values and judgements were the basis of recommendations. The rationale provided by the Working Group for their recommendation against LLLT was the “considerable cost and time burden to be placed on individuals” and that “…clinicians were required to deliver the intervention two to three times a week…” (p. 38). It is unclear why this should be a judgement against LLLT treatment given that most physical therapies including exercise and massage are rarely delivered in a single treatment. From the perspective of time burden, the duration of LLLT treatment would generally be from 20 to 30 min as a maximum. Importantly, the therapeutic benefits of LLLT are based on modulating the underlying pathology and are multifaceted encompassing tissue repair, modulation of inflammation and neural blockade, which are cumulative over several treatments. In contrast, many of the recommended treatments in the Guideline are for symptom management only lasting hours (such as Paracetamol or NSAIDs), or a few weeks at best (such as corticosteroid injection). Massage which also requires repeated treatments is recommended with the same “low” level of evidence as LLLT but is recommended on the basis that it can facilitate more active treatment. If the latter is a reason for inclusion in the Guideline, then the evidence for LLLT is substantial, as several of the studies not included in the Guideline compared LLLT with and without exercise and the majority found that exercise capacity and hence, function is enhanced when combined with LLLT. Moreover, recommended treatments of exercise, dietary advice and psychological support also require considerable time and expenditure.

From a cost perspective, many health professionals (including medical practitioners, physiotherapists, nurses, osteopaths, podiatrists and chiropractors) can administer LLLT as monotherapy or as an adjunct to other therapies. Cost versus benefit is especially relevant when balancing benefits versus harms in the relatively urgent search to find non-opioid alternatives for painful conditions. As LLLT was adjudged to be effective, the resources and costs of LLLT may prove to be less than the long-term burden and costs of drug-taking, especially of opioids or NSAIDs. How much patients value the importance of a treatment may be reflected in their willingness to pay for such treatments. An example cited by Zhang et al. is patients with chronic obstructive pulmonary disease who are willing to pay $13.46 to avoid mild side effects and $67.51 to gain symptom relief [54]. More importantly, a study by Ip et al., which was not in the eight cited RCTs in the Guideline, demonstrated a reduction in the need for knee arthroplasty in patients with KOA treated over 6 years thus with obvious economic benefits [29]. We recognise however that cost-benefit analyses are urgently needed to support this observation for LLLT.

Another of the reasons not to recommend LLLT was said to be the lack of follow-up beyond 3 weeks in the cited studies. This was factually incorrect as at least one of the studies located by the Working Group had a follow-up period of 8 weeks [8]. Moreover, a prospective cohort study over a 6-year period of observation showed that LLLT treatment of knee OA reduced the need for knee replacement [29]. Although this latter was not considered in either the 2018 Guideline or the 2019 meta-analysis, it points to a longer-term benefit of LLLT and should add to a positive cost-benefit analysis.

A fourth issue with the Guideline is the lack of expert input during the development of the recommendations. Balshem and colleagues make the important observation that, “Developing recommendations always requires the opinion of experts….” [55]. To our knowledge, no experts in the field were consulted and none of the members of the Working Group had expert knowledge and understanding of the biological mechanisms and dosing factors of LLLT. Thus, we agree with Bannuru et al. (2014) who suggested that “Guideline developers should word recommendations carefully to avoid losing therapeutic options that may benefit a subset of patients in a field in which few safe and effective treatments are available” [56]. The safety of LLLT has been investigated and confirmed in the published literature [57, 58].

Given the rapidity of technological developments and an exponentially increasing number of publications, our review demonstrates that guidelines can become outdated as soon as they are published without an appropriate mechanism to make any guideline a “living” document. Updating of guidelines appears to be an ad hoc process with an international survey finding that only 53% of guideline developers had in place a formal procedure for deciding when a guideline is out of date [59]. Given the substantial evidence in studies we have cited in this paper, the benefits of LLLT combined with exercise and the findings of the most recent systematic review, we believe that the RACGP Guideline is not reflective of current evidence. This Guideline is not due to be reviewed for another 3 years, meaning that the last cited article on which the LLLT recommendation is based will be 10 years old when the Guideline is next reviewed. Evidence suggests that medical practitioners do not follow guidelines [60] and this paper provides an example of how a guideline which does not contain contemporary information loses validity [61]. The inflexibility of a guideline which is not updated when new information becomes available is a failure of evidence-based practice. The National Institute for Health and Care Excellence (NICE UK), for example, has procedures for updating guidelines and publishing new information on their website which does not require the cost of publishing and which alerts stakeholders to changes when they occur. This could be easily implemented for other guidelines [62].

In a broader context, given the limitations of available treatments and the current imperative to offer non-drug treatments without serious side effects, the conditional “against” recommendation is difficult to understand (Table 1).

**Table 1:**
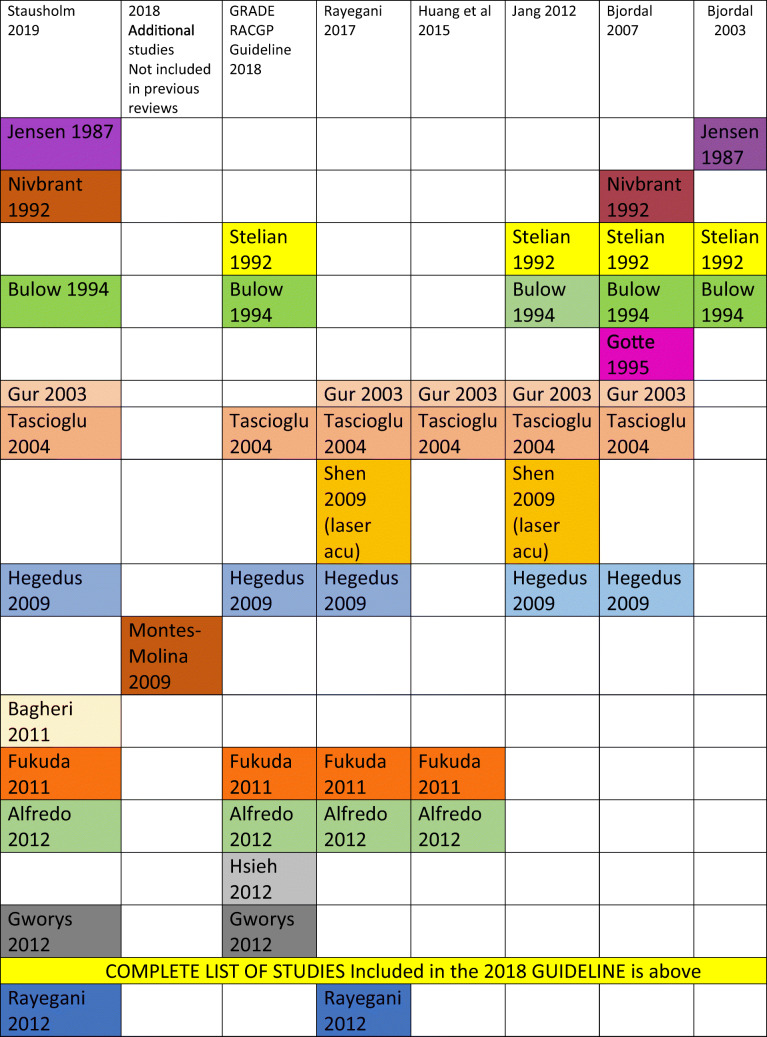

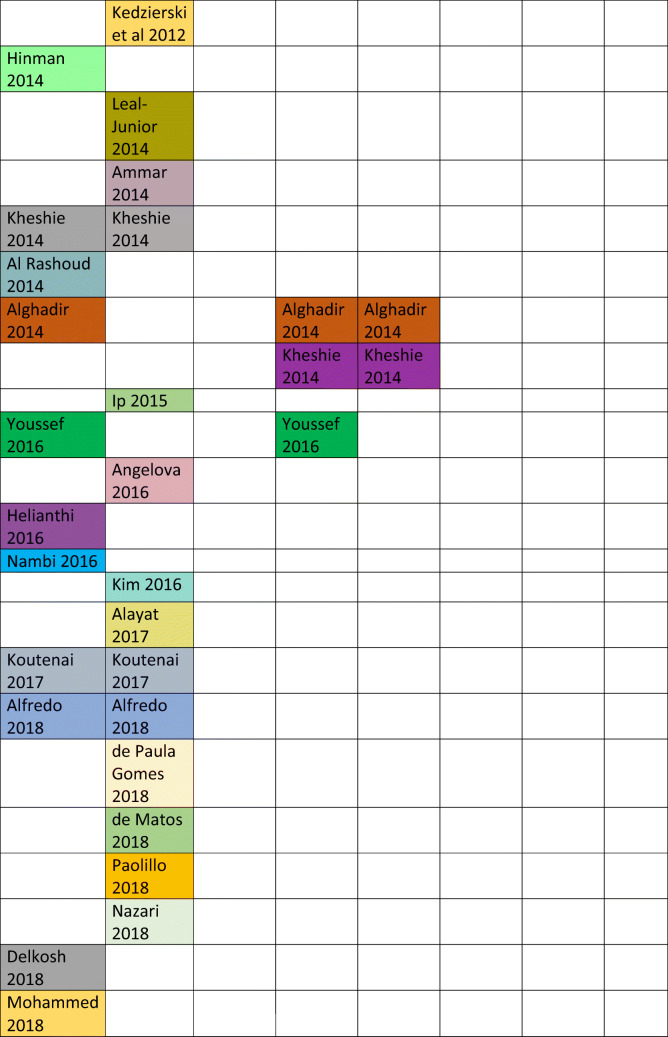
Table of systematic reviews with included studies up to 2019; colour coding relates to studies carried over from one review to another: same colour = same study

## Conclusion

GRADE methodology is generally robust; however, for the process to be effective, a basic tenet is that all the evidence should be reviewed. We believe that the Guideline does not reflect the current evidence for LLLT for knee OA. In contrast, the latest review and meta-analysis [18] strongly supports LLLT for knee OA and the out-of-date Guideline misleads health professionals. Most importantly, patients with KOA who could be receiving a safe, non-invasive, evidence-based, non-drug treatment will not be receiving the benefits of that treatment.

**Fig. 1:**
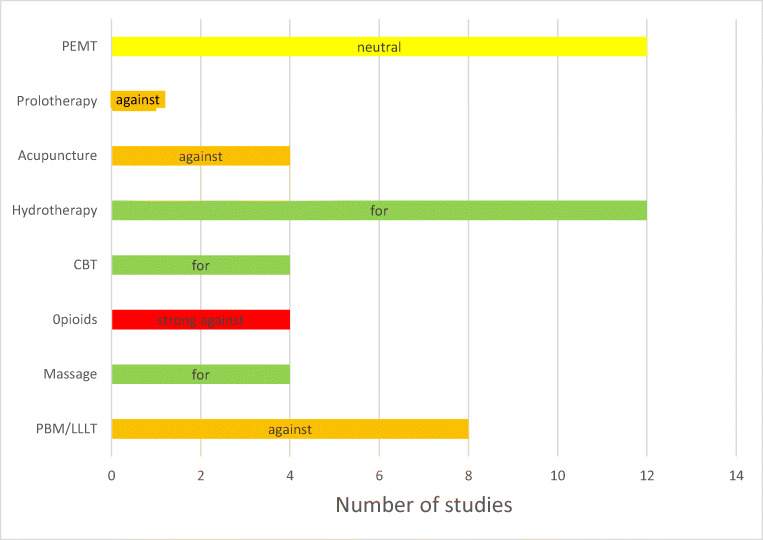
Comparison of the number of studies versus recommendation for the same “low” level of evidence

Given the factors we have identified, we believe that the Guideline should be reviewed immediately and changed from a “conditional against” recommendation for the use of LLLT in KOA to a “for” recommendation to reflect the strength and totality of the published LLLT literature (Fig. 1).

## Data Availability

The data we have are the full texts of references if they are not easily available. We have no mathematical or statistical data in this narrative review. Data are available to the public upon request.
